# No role for standard imaging workup of patients with clinically evident necrotizing soft tissue infections: a national retrospective multicenter cohort study

**DOI:** 10.1007/s00068-023-02414-6

**Published:** 2024-01-23

**Authors:** Sanne R. Brands, Femke Nawijn, Wouter Foppen, Falco Hietbrink

**Affiliations:** Department of Surgery, University Medical Center Utrecht, Utrecht, The Netherlands

**Keywords:** Necrotizing soft tissue infections, Necrotizing fasciitis, Severe necrotizing soft tissue infection, Imaging, Radiology, Treatment delay

## Abstract

**Purpose:**

To assess the diagnostic contribution of different imaging studies to diagnose necrotizing soft tissue infections (NSTIs) and the time to surgery in relation to imaging with the hypothesis that imaging studies may lead to significant delays without being able to sufficiently dismiss or confirm the diagnosis since a NSTI is a surgical diagnosis.

**Methods:**

A retrospective multicenter cohort study of all NSTI patients between 2010 and 2020 was conducted. The primary outcome was the number of cases in which imaging contributed to or led to change in treatment. The secondary outcomes were time to treatment determined by the time from presentation to surgery and patient outcomes (amputation, intensive care unit (ICU) admission, length of ICU stay, hospital stay, and mortality).

**Results:**

A total of 181 eligible NSTI patients were included. The overall mortality was 21% (*n* = 38). Ninety-eight patients (53%) received imaging in the diagnostic workup. In patients with a clinical suspicion of a NSTI, 81% (*n* = 85) went directly to the operating room and 19% (*n* = 20) underwent imaging before surgery; imaging was contributing in only 15% (*n* = 3) by ruling out or determining underlying causes. In patients without a clinical suspicion of a NSTI, the diagnosis of NSTI was considered in 35% and only after imaging was obtained.

**Conclusion:**

In patients with clinically evident NSTIs, there is no role for standard imaging workup unless it is used to examine underlying diseases (e.g., diverticulitis, pancreatitis). In atypical presenting NSTIs, CT or MRI scans provided the most useful information. To prevent unnecessary imaging and radiation and not delay treatment, the decision to perform imaging studies in patients with a clinical suspicion of a NSTI must be made extremely careful.

**Supplementary Information:**

The online version contains supplementary material available at 10.1007/s00068-023-02414-6.

## Background

A necrotizing soft tissue infection (NSTI) is a life-threatening and rare diagnosis. It is an infection known for its fast onset and fulminant disease course, often resulting in sepsis, multiple organ failure, or death [1, 2]. In 2018, the estimated incidence rate of NSTIs in the United States of America was approximately 8.3 to 10.3 cases per 100.000 persons [3]. The incidence rate in the Netherlands is estimated to be much lower, approximately 1.1 to 1.4 cases per 100.000 person years [4]. In addition, the mortality rate of NSTIs remains high worldwide, with recently described mortality rates ranging from 20 to 30 percent [4–7]. A rapid diagnosis followed by immediate surgical debridement is crucial to stop any further progression of the NSTI and therefore lower the chances of unfavorable outcomes [2]. A recent study revealed that surgical debridement within six hours after presentation lowered the mortality rate by almost 50 percent [5]. Thus, a delay in diagnosis can lead to a delay in treatment and therefore a higher mortality rate [8].

To diagnose NSTIs clinicians often rely on a combination of clinical and laboratory findings, which are sometimes combined with imaging and per-operative microscopic tests, such as fresh frozen sections or Gram stains [2, 8]. Pre-operative diagnosis of a NSTI often proves difficult, due to the lack of pathognomonic symptoms and due to varying ways of presentation. The “typical” presentation of a patient with a NSTI consists of pain out of proportion in relation to findings upon physical examination, erythema, swelling, and often sepsis. These symptoms can worsen quickly and––in case of septic shock––may result in death. These “typical” symptoms will quickly raise the suspicion of a NSTI [1, 8–10]. However, some patients present with atypical symptoms, mild progression, and no initial signs of sepsis, resulting in a low suspicion of a NSTI potentially followed by multiple additional tests and imaging studies, and thus a diagnostic delay [1, 8, 9]. Currently, there is no consensus concerning the added diagnostic value of imaging. If the diagnosis is undetermined, radiologic research can be contributing according to the most recent international guideline by Sartelli et al., but exact indications are still missing [7].

Therefore, this study assesses the diagnostic contribution of different imaging studies to diagnose NSTIs and the time to surgery in relation to imaging with the hypothesis that imaging studies may lead to significant delays without being able to sufficiently dismiss or confirm the diagnosis since a NSTI is surgical diagnosis.

## Methods

The institutional review board of the initiating hospital (an academic medical center) approved a waiver (WAG/mb/20/012110) for the retrospective collection of data. This manuscript is written in adherence to the STROBE guideline [11].

### Study design

A retrospective cohort study of all patients with a NSTI presenting to one of the four participating hospitals (one academic tertiary referral medical center and three large peripheral hospitals, located in the province of Utrecht, the Netherlands) between January 1, 2010, and January 1_,_ 2020, was carried out. To identify these patients, a different method per hospital was necessary (Appendix 1). The number of eligible patients, and thus the sample size, was determined by the number of presenting patients to the participating hospitals within the study period. Operative findings (macroscopic findings: swollen tissue, dull grey necrotic tissue, grey fascia, lack of bleeding, small vessel thrombosis, “dishwater” fluid, pus, non-contracting muscle fibers, and a positive “finger test” [12]), microbiological findings (positive Gram stain of fascia), and/or histopathological tissue (full-thickness biopsy positive for NSTI) findings had to confirm the diagnosis of NSTI [7, 12, 13]. Patients younger than 18 years, patients who were lost to follow-up, and patients who received imaging in a different hospital without the full radiology report available were excluded from the study.

### Data collection

Demographic data collected from medical charts consisted of age, sex, obesity (defined as body mass index ≥ 30), diabetes, surgery within 30 days before diagnosis, malignancy, autoimmune disease, heart failure, renal failure, liver failure, and the American Society for Anesthesiologists (ASA) classification. The medical charts consisted of intensive care unit (ICU), operating room, emergency room, ward, and outpatient clinic documents. If the ASA classification was not reported within the medical charts, the ASA classification was determined based on the described comorbidities before the NSTI diagnosis.

Disease-related characteristics collected were the type of NSTI infection (type 1: polymicrobial NSTI and type 2: monomicrobial NSTI) [7], cultured micro-organisms, location of the infection, estimated affected total body surface area (TBSA) (based on the reported extent of the infection and the rule of nine for burn injuries) [14], vital signs at presentation, C-reactive protein (CRP) levels, base excess, and the Laboratory Risk Indicator for Necrotizing Fasciitis (LRINEC)[10, 15]. If reported in the chart or a Sequential Organ Failure Assessment (SOFA) score of two or greater, patients were determined as septic [16]. The LRINEC score was calculated based on the first known laboratory results within twelve hours of presentation.

The imaging characteristics and the conclusion drawn by the treating physician extracted from the charts included the imaging modality (within 24 h before diagnosis), the results of imaging, time to imaging, assumed diagnosis prior to imaging (by analyzing the last mentioned differential diagnosis by the treating physician and the clinical indication for imaging listed within the imaging request form), diagnosis after imaging, contributing and non-contributing imaging, change in treatment, and the physical examination prior to diagnosis (including soft tissue edema, soft tissue gas/crepitus, bullae, erythema, severe pain or tenderness, and skin necrosis) [2, 7, 9]. The imaging studies were categorized to be either clinically contributing or non-contributing. This was done by comparing the pre-imaging diagnosis reported in the charts and the imaging request forms with the post-imaging diagnosis resulting in four different groups. Clinical contributing imaging consisted of the group “no clinical suspicion, NSTI diagnosed with imaging” and the group “clinical suspicion of NSTI, imaging which was used to determine or rule out underlying causes of NSTIs.” Clinical non-contributing imaging was a combination of the groups “no clinical suspicion of NSTI, no signs of NSTI on imaging,” “clinical suspicion of NSTI, confirmed with imaging and in which the indication for imaging was neither excluding NSTI nor determining underlying causes,” and “clinical suspicion of NSTI, no signs of NSTI on imaging” (Appendix 2). Change in treatment was determined by comparing the pre-imaging diagnosis reported in the charts and the imaging request forms and the post-imaging diagnosis and associated treatment plan. It was classified as a change in treatment if the imaging studies caused a change in diagnosis and treatment, which would most likely be the decision to go to the operating room or withdrawal from care. The results of imaging are based on one radiology assessment and were categorized in predetermined categories per imaging modality [2, 7]. Each case was retrospectively reviewed by two researchers by analyzing the medical records of all the included patients. No physical examinations were linked to clinical presentations/suspicion or radiology reports by the researchers.

The primary outcome of this study was the number of cases in which the imaging was contributing to or led to a change in treatment. The secondary outcomes were the time to surgery, with an aim to investigate potential delays between patients undergoing imaging studies and those who did not, and the patient outcomes (amputation, ICU admission, length of ICU stay and hospital stay, and mortality).

### Statistical analysis

Categorical variables were presented with numbers and percentages and continuous variables were presented with standard deviations and means if normally distributed or with interquartile ranges (IQR) and medians if non-normally distributed. Missing data were analyzed using pairwise deletion. Bivariate analyses were used to calculate sensitivity for different imaging modalities and physical examination prior to imaging. Specificity, negative predictive value, and positive predictive value were not determined due to the absence of a control group; Chi-squared tests (for Trend), *t* tests for normally distributed continuous variables, and Mann–Whitney *U* test for non-normally distributed continuous variables were used to evaluate treatment delay and the differences between patients who underwent imaging to diagnose NSTIs and who did not. Outcomes were statistically significant in the case of a two-sided *p*-value < 0.05. Data were analyzed using IBM SPSS statistics 28.0.1.0.

## Results

A total of 186 patients with NSTIs were identified. Five patients were excluded, due to lack of information or were lost to follow-up. Of all patients, 9 (5%) presented initially at a different hospital than treated, of which 5 received imaging; three patients initially presented at a small peripheral hospital outside of the province of Utrecht (one transferred before debridement, two after debridement at a non-participating hospital) and three patients were transferred between the participating hospitals. The median age of these 181 eligible patients was 57 years (IQR 44–71). Most patients were male (64%) and classified as ASA II or III (37% and 34%). The lower extremities were most often affected by the NSTI (43%). The estimated TBSA affected was 4% (IQR 2–6). The baseline characteristics of the included patients are presented in Table 1.

**Table 1:** Baseline characteristics and outcomes of necrotizing soft tissue infection patients

	Total *n *= 181 (100%)	No imaging *n* = 85 (47%)	Imaging *n* = 96 (53%)	*p*-value
Age, median (IQR)	57 (44–71)	59 (45–70)	57 (40–71)	0.481
Male, *n* (%)	116 (64)	52 (61)	64 (67)	0.535
Obesity, *n* (%) ^a^	40 (23)	21 (27)	19 (21)	0.372
Diabetes, *n* (%)	38 (21)	16 (19)	22 (23)	0.584
Surgery < 30 days, *n* (%)	29 (16)	11 (13)	18 (19)	0.316
Malignancy, *n* (%)	28 (16)	10 (12)	19 (19)	0.160
Autoimmune disease, *n* (%)	28 (15)	18 (21)	10 (10)	0.063
Heart failure, *n* (%)	10 (6)	4 (5)	6 (6)	0.752
Renal failure, *n* (%)	8 (4)	6 (7)	2 (2)	0.150
Liver failure, *n* (%)	0 (0)	0 (0)	0 (0)	
ASA classification, *n* (%)				0.796
ASA I	35 (19)	14 (17)	21 (22)	
ASA II	67 (37)	33 (39)	33 (34)	
ASA III	62 (34)	30 (35)	32 (33)	
ASA IV	18 (10)	8 (9)	10 (10)	
Type 1 NSTI, *n* (%)	56 (32)	18 (22)	38 (40)	**0.010**
Type 2 NSTI, *n* (%)	120 (68)	64 (78)	56 (60)	**0.010**
Cultured micro-organism, *n* (%) ^b^				
GAS	81 (46)	44 (54)	37 (39)	0.069
*Clostridium*	9 (5)	2 (2)	7 (7)	0.178
Location of NSTI, *n* (%)				0.146
Head/neck	10 (6)	4 (5)	6 (6)	0.752
Trunk	17 (9)	5 (6)	12 (13)	0.201
Perineum/genitals	48 (27)	20 (24)	28 (29)	0.405
Upper extremities	21 (12)	15 (18)	6 (6)	**0.020**
Lower extremities	78 (43)	37 (44)	41 (43)	1.000
Multiple body areas involved	7 (4)	4 (5)	3 (3)	0.708
Estimated TBSA affected in percentages, median (IQR) ^c^	4 (2–6)	4 (2–7)	3 (2–5)	0.066
CRP in mg/L, median (IQR) ^b^	304 (158–399)	275 (127–396)	316 (176–417)	0.321
Heart rate in beats/minute, median (IQR) ^d^	101 (90–115)	101 (91–112)	102 (90–119)	0.619
Systolic blood pressure in mmHg, mean (SD) ^c^	117 (23)	118 (24)	117 (22)	0.869
Sepsis upon admission, n (%) ^e^	58 (34)	30 (38)	28 (30)	0.335
LRINEC score, median (IQR) ^f^	7 (5–9)	7 (5–9)	7 (5–8)	0.610
Base excess, median (IQR) ^g^	− 12 (− 36–− 3)	− 13 (− 45–− 3)	− 9 (− 22–− 3)	0.343
Time presentation to surgery hours, median (IQR) ^h^	7 (3–26)	5 (3–26)	12 (4–31)	0.051
Amputation, *n* (%)	22 (12)	13 (16)	9 (10)	0.258
ICU admission, *n* (%)	138 (76)	64 (75)	74 (77)	0.862
Length of ICU stay in days, median (IQR) ^i^	4 (2–12)	6 (1–15)	4 (2–9)	0.630
If survived, length of hospital stay in days, median (IQR) ^i^	25 (15–44)	26 (17–49)	23 (13–41)	0.343
Mortality, n (%)	38 (21)	21 (25)	17 (17)	0.276

Bold values indicate that *p* < 0.05

^a^ 10 missings, ^b^ 5 missings, ^c^ 9 missings, ^d^ 7 missings, ^e^ 8 missings, ^f^ 35 missings, ^g^ 85 missings, ^h^ 14 missings, ^i^ 1 missing

*ASA* American Society for Anesthesiologists, *CRP* C-reactive protein, *GAS* Group A Streptococcus, *ICU* intensive care unit, *IQR* interquartile range, *LRINEC* laboratory risk indicator for necrotizing fasciitis, NSTI necrotizing soft tissue infection, *TBSA* total body surface area,

### Clinical suspicion vs. no clinical suspicion

**Fig. 1:**
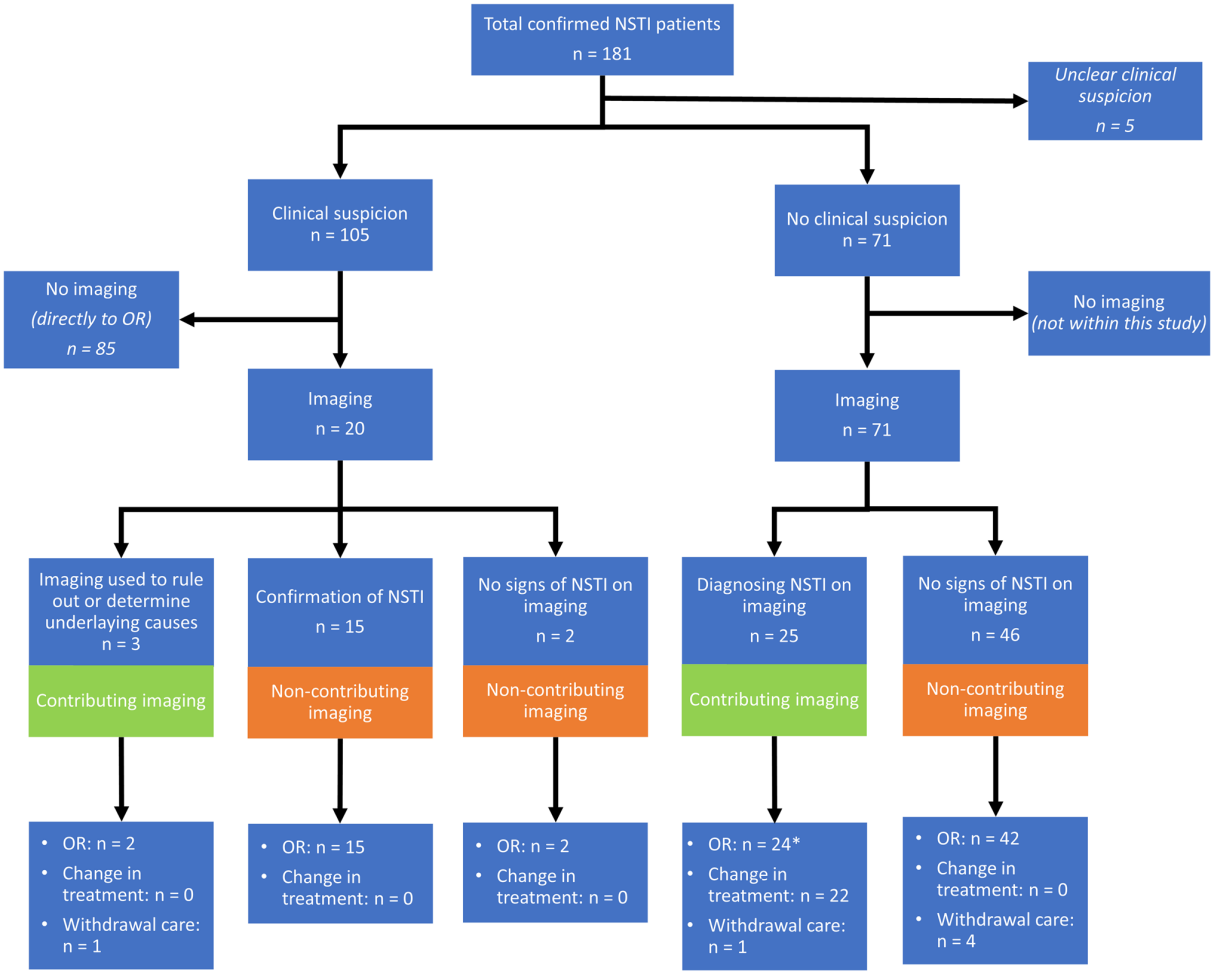
Flowcharts of necrotizing soft tissue infection patients divided into contributing and non-contributing imaging. Legend: *no debridement: *n* = 2; no complete debridement: *n* = 1. *NSTI* necrotizing soft tissue infection; *OR* operating room

A total of 105 (60%) patients had an evident clinical suspicion for NSTI upon presentation (were either brought directly to operating room or the treating physician reported NSTI clearly as diagnosis already before imaging was requested). In patients with a clinical suspicion of a NSTI (*n* = 105, 60%; Fig. 1 and Appendix 3), the most common location of NSTIs were the lower extremities (*n* = 42, 44%) followed by perineum/genitals (*n* = 31, 30%) and the upper extremities (*n* = 17, 16%). The most common location in patients without a clinical suspicion (*n* = 71, 40%) was the lower extremities (*n* = 34, 48%), followed by the perineum/genitals (*n* = 14, 20%) and the trunk (*n* = 11, 16%). Of the 10 patients with a NSTI of the head/neck, two patients developed descending necrotizing mediastinitis (20%). Patients without a clinical suspicion of a NSTI before imaging had suspected diagnoses such as cellulitis (*n* = 6, 8%), sepsis without a clear cause (*n* = 9, 12%), abscesses (*n* = 25, 35%), deep vein thrombosis (*n* = 10, 13%), or other unrelated diagnoses (*n* = 19, 27%). Two patients had a differential diagnosis in which several categories occurred (DVT and abscess; DVT and space-consuming process). Patients with a clinical suspicion of a NSTI had significantly more infections located in the upper extremities (16% vs. 6%; *p* = 0.036) and significantly less infections located in the trunk (5% vs. 16%; *p* = 0.029). In patients with a clinical suspicion of a NSTI, the median time from presentation to surgery was 5.5 h (IQR 3–25), which is significantly lower than 15 h (IQR 4.5–31.5) for patients without a clinical suspicion (*p* = 0.015) (Appendix 3). The median time from presentation to surgery was 5 h (IQR 3–25.5) for patients who went directly to the operating room (OR) after clinical suspicion, which is shorter compared to 6 h (IQR 3–20.5) in patients who underwent imaging first (*p* = 0.933).

### Imaging in NSTI patients

In a total of 96 patients, 53% underwent imaging studies during the diagnostic workup (Table 1). In 73% of patients, surgeons were already involved before imaging. Type 1 NSTIs were significantly more common in patients receiving imaging than patients without imaging (*n* = 38, 40% vs. *n* = 18, 22%; *p* = 0.010).

Patients undergoing imaging had significantly less often a NSTI of the upper extremities compared to patients without imaging (*n* = 6, 6% vs. *n* = 15, 18%; *p* = 0.020). A median of two hours (IQR 1–4) elapsed between presentation and the imaging studies. The median time from presentation to surgery for all the included patients was 7 h (IQR 3–26) (Table 1). The median time from presentation to surgery was 12 h (IQR 4–31) in patients receiving imaging to diagnose a NSTI, which is more than twice as high as 5 h (IQR 3–26) in patients not receiving imaging to diagnose a NSTI (*p* = 0.051). There were no other differences between outcomes in the group with and without imaging. The overall mortality was 21% (*n* = 38). After adjusting for ASA classification and estimated TBSA, the use of imaging was not significantly associated with mortality (*p* = 0.186). The majority of patients were initially admitted by surgical specialties (surgeons, urologists, and ear, nose, and throat (ENT) doctors) (79%). Patients admitted by non-surgical specialties did not undergo significantly more often imaging (*n* = 18, 7%. *n* = 78, 55%; *p* = 0.466). However, the median time from presentation to imaging was more than twice as high in patients admitted by non-surgical specialties compared to patients admitted by surgeons (4 (2.5–38) vs. 1.5 (1–3.5); *p* =  < 0.001). Other patients were admitted by internal medicine specialists (18%), ophthalmologists (1.1%), plastic surgeons (0.6%), cardiologists (0.6%), oral surgeons (0.6%), and orthopedic surgeons (0.6%).

A total of 109 imaging studies were performed (Table 2). Forty-five patients received an ultrasound (41%), 45 received a computed tomography scan (CT scan) (41%), fourteen received an X-radiography (X-ray) (13%), and five received a magnetic resonance imaging scan (MRI scan) (5%). Eleven patients who underwent an ultrasound (US) (20%) also received another imaging modality in addition. Eight ultrasounds were followed by a CT scan and three ultrasounds were followed by an MRI scan. Furthermore, three patients (21%) received another imaging modality in addition to the X-ray. Two X-rays were followed by a CT scan and one X-ray was followed by an ultrasound. Subcutaneous emphysema/gas (*n* = 24, sensitivity: 53%) and soft tissue edema (*n* = 27, sensitivity 60%) were findings most seen on CT scans. All the MRI scans showed soft tissue edema (*n* = 5, sensitivity: 100%). The most common finding of ultrasounds was soft tissue edema (*n* = 37, sensitivity: 80%) and indifferent findings (*n* = 8, sensitivity: 57%) for X-rays (Table 2).

**Table 2:** Findings of imaging studies to diagnose necrotizing soft tissue infections

All imaging studies performed in 96 patients, *n* (%)	109 (100)
All imaging studies resulting in a change in treatment, *n* (%) ^a^	25 (23)
Time from presentation to imaging in hours for all imaging studies, median (IQR)	2 (1–4)
Total CT scans performed, *n* (%)	45 (41)
CT scans resulting in a change of treatment, *n* (%) ^a^	17 (38)
Time from presentation to imaging in hours for CT scans, median (IQR) ^a^	3 (1.5–12.5)
Findings of CT scan, *n* (%):	
Fascial edema/fluid collections	2 (4)
Fascial thickening	1 (2)
Fascial gas	4 (9)
Subcutaneous emphysema/subcutaneous gas	24 (53)
Soft tissue edema	27 (60)
Muscle induration	4 (9)
Indifferent	5 (11)
Total MRI scans performed, *n* (%)	5 (5)
MRI scans resulting in a change of treatment, *n* (%) ^a^	3 (60)
Time from presentation to imaging in hours for MRI scans, median (IQR)	3 (2.5–3.5)
Findings of MRI scan, *n* (%):	
Fascial edema/fluid collections	1 (20)
Fascial thickening	1 (20)
Fascial gas	0
Subcutaneous emphysema/subcutaneous gas	1 (20)
Soft tissue edema	5 (100)
Indifferent	0
Total ultrasounds performed, *n* (%)	45 (41)
Ultrasounds resulting in a change of treatment, *n* (%) ^a^	4 (9)
Time from presentation to imaging in hours for ultrasounds, median (IQR)	2 (1–4)
Findings of ultrasound, *n* (%)	
Fascial thickening	1 (2)
Fascial gas	1 (2)
Subcutaneous emphysema/subcutaneous gas	12 (27)
Soft tissue edema	36 (80)
Muscle induration	1 (2)
Indifferent	6 (13)
Total X-rays performed, *n* (%)	14 (13)
X-rays resulting in a change of treatment, *n* (%) ^a^	1 (7)
Time from presentation to imaging in hours for X-rays, median (IQR)	1.5 (0.5–2.5)
Findings of X-rays, *n* (%):	
Subcutaneous emphysema/subcutaneous gas	3 (21)
Indifferent	8 (57)
No abnormalities	3 (21)

^a^ 1 missing

*CT* computed tomography, *IQR* interquartile range, *MRI* magnetic resonance imaging, *US* ultrasound, X-ray X-radiography

### Correlation of physical examination and imaging findings

The findings during physical examination were available for 179 patients (99%). A total of 118 patients presented with edema at physical examination (66%), and 58 of those patients received imaging (49%). When soft tissue edema was detected on the CT scan (*n* = 28, 61%), 36% (*n* = 10) of the edema was not priorly seen at physical examination. In the case of edema found on the MRI scan (*n* = 5, 100%), 40% (*n* = 2) of the soft tissue edema was also seen at physical examination and in the case of the ultrasound (*n* = 35, 80%), 71% (*n* = 25) of the soft tissue edema was seen at physical examination. A total of 18 patients presented with gas at physical examination (10%), and 14 of those patients received imaging (78%). CT scans detected clinically occult gas in 52% (*n* = 24) of the patients. In 21% (*n* = 5) of the patients, it was also seen at physical examination. In the case of gas on the MRI scan (*n* = 1, 20%), it was not noted at physical examination, and in the case of gas detected with the ultrasound (*n* = 12, 27%), 25% (*n* = 3) was seen at physical examination. When gas was detected with the X-ray (*n* = 3, 21%), none of the patients noted it during physical examination.

### Clinical consequences of imaging

In 69% of the patients (*n* = 63), imaging studies were considered non-contributing (Fig. 1, Table 3, and Appendix 2). In patients with a clinical suspicion of a NSTI, imaging was more non-contributing compared to patients without a clinical suspicion (*n* = 17, 85% vs. *n* = 46, 65%; *p* = 0.104). Polymicrobial NSTIs were significantly more common in patients with imaging contributing to clinical care (*n* = 16, 59% vs. *n* = 19, 31%; *p* = 0.018), while monomicrobial NSTIs were significantly more common in patients receiving non-contributing imaging (*n* = 43, 69%, vs. *n* = 11, 41%; *p* = 0.018). In addition, the group undergoing non-contributing imaging had significantly less often a GAS NSTI compared to the group undergoing contributing imaging (*n* = 30, 48% vs. *n* = 5, 19%; *p* = 0.010). Thereby, patients undergoing contributing imaging had more often a *Clostridium* NSTI compared to patients undergoing non-contributing imaging (*n* = 5, 19%, vs. *n* = 2, 3%; *p* = 0.025). Between these groups, a significant difference was found in terms of change of treatment (*n* = 0, 0%) in the non-contributing group compared to contributing group (*n* = 22, 79%) (*p* < 0.01) (Table 3), of which 21 patients went directly to the OR after the radiology results and there was withdrawal of care in one case based on patient’s and family’s wishes.

**Table 3:** Differences in baseline characteristics and outcomes between patients undergoing contributing and non-contributing imaging

	Non-contributing imaging ^a^ *n* = 63 (69%)	Contributing imaging ^a^ *n* = 28 (31%)	*p-*value*
Type 1 NSTI, *n* (%) ^b^	19 (31)	16 (59)	**0.018**
Type 2 NSTI, *n* (%) ^b^	43 (69)	11 (41)	**0.018**
Micro-organism, n (%)^b^			
GAS	30 (48)	5 (19)	**0.010**
*Clostridium*	2 (3)	5 (19)	**0.025**
Location of NSTI, *n* (%)			0.445
Head/neck	4 (6)	2 (7)	1.000
Trunk/perineum	22 (35)	14 (50)	0.245
Extremities	36 (57)	11 (39)	0.172
Multiple body areas involved	1 (2)	1 (4)	0.523
Sepsis upon admission, *n* (%) ^c^	15 (25)	12 (44)	0.081
Estimated TBSA affected in percentages, median (IQR) ^d^	3 (2–5)	3 (2–8)	0.527
CRP in mg/L, median (IQR)^b^	288 (119–368)	318 (186–426)	0.275
Heart rate in beats/minute, median (IQR) ^e^	100 (89–116)	103 (89–119)	0.921
Systolic blood pressure in mmHg, mean (SD)^c^	115 (21)	123 (24)	0.169
LRINEC score, median (IQR) ^f^	7 (3–8)	7 (6–9)	0.124
Base excess, median (IQR) ^g^	-9 (-22−3)	-12 (-29–1)	0.645
Time presentation to surgery hours, median (IQR) ^h^	15 (4–32)	7 (4–23)	0.739
Causing a change in treatment, *n* (%)	0 (0)	22 (79)	** < 0.001**
Amputation, *n* (%) ^i^	5 (8)	4 (15)	0.446
ICU admission, *n* (%)	50 (79)	20 (71)	0.428
Length of ICU stay in days, median (IQR) ^j^	5 (2–8)	3 (2–9)	0.952
If survived, length of hospital stay in days, median (IQR) ^k^	23 (13–40)	23 (13–58)	0.644
Mortality, *n* (%)	8 (13)	8 (29)	0.080

Bold values indicate that *p* < 0.05

^a^ 5 missings, ^b^ 2 missings, ^c^ 3 missings, ^d^ 7 missings, ^e^ 4 missings ^f^ 16 missings, ^g^ 50 missing, ^h^ 9 missings, ^i^ 4 missings, ^j^ 23 missings, ^k^ 1 missing

*CRP* C-reactive protein, *GAS* Group A streptococcus, *ICU* intensive care unit, *IQR* interquartile range, *LRINEC* laboratory risk indicator for necrotizing fasciitis, *NSTI* necrotizing soft tissue infection, *TBSA* total body surface area

^*^P-values of comparison between contributing and non-contributing imaging. Clinical contributing imaging consist of the group “no clinical suspicion, NSTI diagnosed with imaging.” Clinical non-contributing imaging is a combination of the groups “no clinical suspicion of NSTI, no signs of NSTI on imaging,” “clinical suspicion of NSTI confirmation on imaging,” and “clinical suspicion of NSTI, no signs of NSTI on imaging”

## Discussion

### Principal findings

More than half of all the NSTI patients within this study underwent imaging studies; however, imaging studies were contributing to the diagnosis in only 31% and led to a change in treatment in only 24%. In patients with clinically suspected NSTIs, imaging studies were contributing if used to determine or rule out underlying causes of NSTIs, for example, diverticulitis and pancreatitis. In all other cases, there was no place for imaging. In the case of no evident clinical suspicion of a NSTI, imaging aided the diagnosis in 35% of the cases.

### Strengths and limitations

This is the first study assessing the potential delay caused by imaging studies in NSTI patients in a fairly large cohort of NSTI patients. However, the results of this study must be interpreted within the context of its limitations due to the study’s retrospective nature. First, the imaging findings described in this study were based on the radiology reports written by the radiologist reporting the imaging at the time the patient presented. There was no reevaluation of the radiology imaging itself for this study; therefore, additional findings such as sarcopenia could not be extracted and analyzed. Furthermore, variation in the extensiveness of reporting the findings within the radiology reports was inevitable. Second, the physical examination findings and estimated TBSA affected used for this study were based on findings reported in the medical charts by the physician who examined the patients upon presentation. Third, the retrospective aspect of this study also resulted in missing data, especially the LRINEC score, therefore resulting in information bias. Fourth, the aspect of this study potentially resulted in confounding by indication.

### Comparison with other studies: Radiological findings of NSTIs

A recent meta-analysis by Fernando et al. investigated the diagnostic accuracy of imaging to diagnose a NSTI (Table 4) [2]. Based on those results, they discouraged the use of plain X-radiography, which is further supported by this study, since 21% of the patients required additional imaging and the results were indifferent in 57% of the cases. Furthermore, Fernando et al. warned about the logistical challenges of CT and MRI scans which can cause delays by waiting on the scan itself and/or the interpretation of the radiologist [2, 9]. Different studies present different characteristics of importance for each imaging modality. Fascial thickening is the most crucial feature to diagnose a NSTI on CT or MRI scan, which could represent necrosis of the fascia [7, 17–22]. Other important features include subcutaneous gas, subcutaneous edema/soft tissue edema, fluid collections, and muscle induration (Table 4) [2, 7, 9, 17–22]. The most contributing imaging features in the present study were subcutaneous gas on CT scan and soft tissue edema on ultrasound, CT scan, and MRI scan. However, gas is a phenomenon that is not commonly seen (5–24% of the cases), since only NSTIs caused by bacteria that produce gas, such as *Clostridium*, and might not be present in the early stages of NSTIs caused by *Clostridium* [7, 9, 10, 23]. Soft tissue edema is also seen in other soft tissue infections and does not have to represent a necrotizing infection [24]. Fascial thickening remains one of the most specific findings on a CT or MRI scan for NSTIs [7]. It is also seen in eosinophilic fasciitis; however, the clinical presentation differs from a necrotizing soft tissue infection [22]. The importance of awareness of this finding is of the upmost importance for radiologists and physicians. An atypical presentation (e.g., clinically unsuspected NSTIs) and therefore unclear requests for imaging studies can cause doubts and uncertainties for both radiologists and surgeons, which can cause a greater diagnostic delay and therefore prolong the time to treatment (Table 5).

**Table 4:** Results of sensitivity and specificity of findings on imaging in necrotizing soft tissue infection patients in two recent meta-analyses

	Fernando et al. [2]		Kwee et al. [17]	
X-ray		Sensitivity 49% Specificity 94%		
CT-scan	Signs of fascial edema, fascial enhancement, or facial gas	Sensitivity 94% Specificity 77%	Subcutaneous gas	Sensitivity 49% Specificity 93%
			Subcutaneous edema	Sensitivity 98% Specificity 11%
			Fluid collections	Sensitivity 35% Specificity 88%
MRI scan			Hyperintensity of fascia	Sensitivity 86% Specificity 65%
			Thickening of fascia	Sensitivity 62% Specificity 87%
			Multicompartmental involvement	Sensitivity 76% Specificity 71%

*CT* computed tomography, *MRI* magnetic resonance imaging, *X-ray* X-radiography

**Table 5:** Key points for both radiologists and surgeons

	Radiologists	Surgeons
Indication to perform imaging studies	- To rule out underlying illness potentially causing the NSTI - To rule out non-NSTI differential diagnoses	
Signs of NSTIs	On imaging: - Pathognomonic findings: Fascial thickening - Non-pathognomonic findings: Soft tissue edema, Subcutaneous emphysema/ subcutaneous gas	At clinical presentation: - Pathognomonic findings: None - Non-pathognomonic findings: Soft tissue edema, erythema, severe pain or tenderness, skin necrosis, crepitus, tachycardia, fever, hypotension (9)
Key points	- When imaging is indicated, it is important to perform imaging as quickly as possible to prevent delay - When imaging is necessary, CT or MRI scans are preferred - When fascial thickening appears, rapid communication between radiologists and surgeons is needed	- When a NSTI is suspected, act as quickly as possible to prevent delay - Only use imaging when necessary - Relay to the radiologist the reasons for the urgency of the imaging - Rapid debridement will decrease mortality and is the most important treatment (5) - Be aware of an atypical presentation, e.g., slow onset of atypical symptoms without sepsis (9)

### Imaging and NSTI types

GAS NSTIs are known to cause more often the “typical” fulminant NSTI compared to polymicrobial NSTIs [4]. Due to this typical presentation and rapid progression, there is often more awareness of the possibility of a NSTI resulting in the decision to perform a surgical exploration. In this study, patients with monomicrobial infections received significantly less often imaging studies**.** Due to this awareness, reduced diagnostic delays, and a relatively healthier population of patients, the mortality of this group is lower compared to polymicrobial. Polymicrobial infections, also known as type 1 NSTIs, are known for their atypical presentation [7, 9]. However, these types of infections are less fulminant [4, 9]. These types of NSTIs were more common in patients receiving contributing imaging and did not result in significant delays to treatment. This suggests that imaging could be beneficial within this group, due to its atypical presentation. However, it should be noted that it resulted in a diagnosis in only 35% of these patients and the use of imaging within this group should still be weighed carefully. Furthermore, *Clostridium* NSTIs were significantly more often seen in the group of contributing imaging; however, since *Clostridium* NSTIs are rare in the Netherlands, this group was very small within our study.

### Clinical implications

Recent studies of our study group and Gelbard et al.’s showed that surgical debridement within 12 h significantly lowered the mortality rate, and surgical debridement within 6 h might even decrease mortality further [5, 25]. As seen in this study, the median time from presentation to surgery in patients who received imaging to diagnose a NSTI was 12 h, which is more than twice as high as the 5 h in the group not receiving imaging. However, the treatment delay of several hours between clinically suspected NSTIs and clinically non-suspected NSTIs cannot be contributed to imaging alone, but more likely to lack of clinical suspicion and evading strategies. Nonetheless, urgent surgical intervention is still needed to prevent mortality and reduce severe morbidity [4, 5, 9, 26, 27]. However, the mortality between patients undergoing and not undergoing imaging studies was not significantly higher. This may arise from an already-low overall mortality within this cohort of 21% or the relatively small sample size compared to the meta-analyses previously mentioned [5, 15]. In the present study, imaging was performed in 19% of patients with a typical presentation/clinical suspicion of NSTI and appeared to be contributing in only 15% by ruling out or conforming underlying diseases. Therefore, there is no role for standard imaging workup as it is likely to delay urgent surgical debridement in patients with a typical presentation of NSTI. As previously mentioned, imaging can help determine or rule out underlying causes such as descending mediastinitis, diverticulitis, and pancreatitis.

### Unanswered questions and future research

In this study, imaging had limited contribution to the diagnosis of patients with a clinically evident NSTI, mostly because of its typical presentation. However, it is more complicated to recognize patients with an atypical presentation and a clinically less evident NSTI. Further research should investigate this atypical presentation and clarify the diagnostic workup for these patients, ideally developing a scoring system to determine whether imaging contributes or whether patients should go directly to the OR for investigation. In addition, given the retrospective aspect of the study, this study could be conducted in a large prospective cohort with patients in which NSTI was suspected but ruled out to increase the impact of the evaluation of the diagnostic value of imaging.

## Conclusion

In patients with clinically evident NSTIs, there is no role for standard imaging workup unless it is used to examine potential underlying diseases (e.g., descending mediastinitis, diverticulitis, and pancreatitis). In patients with an atypical presenting NSTI, CT scan or MRI scan provided the most useful information. Given the need for urgent surgical intervention to prevent mortality and reduce severe morbidity, the decision to perform imaging studies with a clinical suspicion of a NSTI must be made extremely careful to prevent unnecessary imaging and radiation, but most importantly not delay treatment.

### Supplementary Information

Below is the link to the electronic supplementary material.

Supplementary file1 (DOCX 15 KB)

Supplementary file2 (DOCX 25 KB)

Supplementary file3 (DOCX 25 KB)

## Data Availability

The dataset used and analyzed during the current study is available from the corresponding author on reasonable request.

## Abbreviations

ASA: American Society for Anesthesiologists
CRP: C-reactive protein
CT: Computed tomography
ENT: Ear, nose, throat
GAS: Group A streptococcus
ICU: Intensive care unit
IQR: Interquartile range
LRINEC: Laboratory risk indicator for necrotizing fasciitis
MRI: Magnetic resonance imaging
NSTI: Necrotizing soft tissue infection
OR: Operating room
SOFA: Sequential organ failure assessment
TBSA: Total body surface area
X-ray: X-radiography
