# Supplementary material for: No role for standard imaging workup of patients with clinically evident necrotizing soft tissue infections: a national retrospective multicenter cohort study
Source: Eur J Trauma Emerg Surg. 2024 Jan 23;50(3):875–85. doi: 10.1007/s00068-023-02414-6 (PMC11249592; doi:10.1007/s00068-023-02414-6)
Supplement: Supplementary file 1 — Supplementary file1 (DOCX 15 KB) [file 68_2023_2414_MOESM1_ESM.docx]

**Appendix 1: Methods of identifying patients with necrotizing soft tissue infections**

| University Medical Center Utrecht | Jan 2010 – Jan 2013 | Patient sought using the International Code for Disease (ICD) 10 for necrotizing fasciitis (M72.6) |
| --- | --- | --- |
|  | Jan 2013 – Dec 2019 | Prospective database of patients with necrotizing soft tissue infection |
| St. Antonius Hospital | Jan 2010 – Sept 2016 | Patients identified using search terms necrotizing fasciitis, Fournier gangrene, myonecrosis in three databases:   - Rare disease list kept by intensive care department - The consulting system of the microbiology department - The microbiology laboratory information management system for documented positive fascia cultures |
|  | Oct 2016 – Dec 2019 | Patient sought using the International Code for Disease (ICD) 10 for necrotizing fasciitis (M72.6) and Fournier gangrene (N49.3) and the Surgical Diagnosis Treatment Combination (DBC) codes for necrotizing fasciitis (164), soft tissue infections (160), large wounds (282) and Fournier gangrene (068 and 098) |
| Diakonessenhuis | Jan 2010 – Dec 2019 | Patient sought using the International Code for Disease (ICD) 10 for necrotizing fasciitis (M72.6) and Fournier gangrene (N49.3) and the Surgical Diagnosis Treatment Combination (DBC) codes for necrotizing fasciitis (164), soft tissue infections (160), large wounds (282) and Fournier gangrene (068 and 098) |
| Meander Medical Center | Jan 2010 – Dec 2019 | Patient sought using the Surgical Diagnosis Treatment Combination (DBC) codes for necrotizing fasciitis (164), soft tissue infections (160), large wounds (282) and Fournier gangrene (068 and 098) |
