# Supplementary material for: No role for standard imaging workup of patients with clinically evident necrotizing soft tissue infections: a national retrospective multicenter cohort study
Source: Eur J Trauma Emerg Surg. 2024 Jan 23;50(3):875–85. doi: 10.1007/s00068-023-02414-6 (PMC11249592; doi:10.1007/s00068-023-02414-6)
Supplement: Supplementary file 2 — Supplementary file2 (DOCX 25 KB) [file 68_2023_2414_MOESM2_ESM.docx]

## **Appendix 2: Formation of groups “contributing and non-contributing imaging” in NSTI patients**

|  | Contributing imaging | | Non-contributing imaging | | |  |
| --- | --- | --- | --- | --- | --- | --- |
|  | *No clinical suspicion, NSTI diagnosed with imaging* | *Imaging to rule out or determine underlying causes* | *No clinical suspicion, No signs of NSTI on imaging* | *Clinical suspicion, confirmation on imaging* | *Clinical suspicion, no signs of NSTI on imaging* |  |
| Total n(%) ^a^ | 25(28) | 3(3) | 46(51) | 15(17) | 2(2) |  |
| Type 1 NSTI, n(%) ^b^ | 14 (58) | 2(67) | 11(24) | 8(53) | 0(0) |  |
| Type 2 NSTI, n(%) ^b^ | 10(42) | 1(33) | 34(76) | 7(47) | 2(100) |  |
| Total imaging studies, n(%) | 33(32) | 4(4) | 46(44) | 17(16) | 4(4) |  |
| Number of CT scans, n(%) | 16(48) | 3(75) | 16(35) | 7(41) | 1(25) |  |
| Number of MRI scans, n(%) | 3(9) | 0(0) | 0(0) | 2(12) | 0(0) |  |
| Number of Ultrasounds, n(%) | 12(36) | 1(25) | 20(43) | 8(47) | 1(25) |  |
| Number of X-rays, n(%) | 2(6) | 0(0) | 10(22) | 0(0) | 2(50) |  |
| Micro-organism |  |  |  |  |  |  |
| - GAS, n(%) ^b^ | 5(21) | 0(0) | 22(49) | 6(40) | 2(100) |  |
| - *Clostridium,* n(%) ^b^ | 5(21) | 0(0) | 1(2) | 1(7) | 0(0) |  |
| Location of NSTI   - Head/ neck - Trunk/ Perineum - Extremities - Multiple body areas involved | 2(8)  13(52)  9(36)  1(4) | 0(0)  1(33)  2(67)  1(2) | 4(9)  12(26)  29(63)  1(2) | 0(0)  10(67)  5(33)  0(0) | 0(0)  0(0)  2(100)  0(0) |  |
| Sepsis upon admission, n(% ) ^c^ | 11(46) | 1(33) | 11(24) | 4(29) | 0(0) |  |
| Estimated TBSA affected in percentages, median(IQR) ^d^ | 3(2-7) | 3(2-X) | 4(2-5) | 3(2-5) | 5(4-X) |  |
| LRINEC score, median(IQR) ^e^ | 7(6-9) | 8(6-X) | 6(3-8) | 8(7-9) | 7(6-X) |  |
| Base-excess, median(IQR) ^f^ | -13(-32 - -2) | -6(-12 - X) | -8(-18 - -2) | -10(-34 - -2) | - |  |
| Time presentation to surgery hours, median(IQR) ^g^ | 6.5(4-23) | 68(15-X) | 17(5-36) | 5.5(2.5-16) | 17(2.5-X) |  |
| Causing a change in treatment, n(%) | 22(88) | 0(0) | 0(0) | 0(0) | 0(0) |  |
| Amputation, n(%) ^b^ | 4(17) | 0(0) | 3(7) | 2(13) | 0(0) |  |
| ICU admission, n(%) | 19(76) | 1(33) | 37(80) | 12(80) | 1(50) |  |
| Length of ICU stay in days, median(IQR) ^h^ | 3(2-8) |  | 5(2-10) | 3(1-7) |  |  |
| If survived, Length of hospital stay in days, median(IQR) ^i^ | 23(13-57) | 86(18-X) | 22(12-41) | 25(14-40) |  |  |
| Mortality, n(%) | 7(28) | 1(33) | 7(15) | 1(7) | 0(0) |  |
| *GAS= Group A Streptococcus, ICU= Intensive Care Unit, IQR= Interquartile range, LRINEC= Laboratory risk indicator for necrotizing fasciitis, NSTI= Necrotizing soft tissue infection, TBSA= Total body surface area. ^a^: 5 missings, ^b^: 2 missings, ^c:^ 3 missings, ^d^: 7 missings, ^e^: 16 missings, ^f^: 49 missings, ^g^: 8 missings, ^h^: 23 missings, ^i:^ 1 missing* | | | | | | |
