# Supplementary material for: No role for standard imaging workup of patients with clinically evident necrotizing soft tissue infections: a national retrospective multicenter cohort study
Source: Eur J Trauma Emerg Surg. 2024 Jan 23;50(3):875–85. doi: 10.1007/s00068-023-02414-6 (PMC11249592; doi:10.1007/s00068-023-02414-6)
Supplement: Supplementary file 3 — Supplementary file3 (DOCX 25 KB) [file 68_2023_2414_MOESM3_ESM.docx]

# **Appendix 3: Differences in baseline characteristics and outcomes between clinical suspected NSTI patients and non-clinical suspected NSTI patients**

|  | Clinical suspicion ^a^  n = 105(60%) | Non-clinical suspicion ^a^  n = 71(40%) | *p*-value |
| --- | --- | --- | --- |
| Age, median(IQR) | 59(45-70) | 57(39-71) | 0.510 |
| Male, n(%) | 69(66) | 43(61) | 0.525 |
| Obesity, n(%) ^b^ | 26(26) | 14(20) | 0.462 |
| Diabetes, n(%) | 20(19) | 17(24) | 0.456 |
| Surgery <30 days, n(%) | 13(12) | 14(20) | 0.205 |
| Malignancy, n(%) | 12(11) | 16(23) | 0.059 |
| Auto-immune disease, n(%) | 20(19) | 8(11) | 0.209 |
| Heart failure, n(%) | 5(5) | 5(7) | 0.527 |
| Renal failure, n(%) | 6(6) | 2(3) | 0.477 |
| Liver failure, n(%) | 0(0) | 0(0) |  |
| ASA classification, n(%) |  |  | 0.543 |
| - ASA I | 16(15) | 17(24) |  |
| - ASA II | 41(39) | 24(34) |  |
| - ASA III | 37(35) | 23(32) |  |
| - ASA IV | 11(11) | 7(10) |  |
| Type 1 NSTI, n(%) ^a^ | 28(28) | 25(36) | 0.242 |
| Type 2 NSTI, n(%) ^a^ | 74(73) | 44(64) | 0.242 |
| Cultured micro-organism, n(%) |  |  |  |
| - GAS ^a^ | 52(51) | 27(39) | 0.160 |
| - *Clostridium* ^a^ | 3(3) | 6(9) | 0.160 |
| Location of NSTI, n(%) |  |  | **0.021** |
| - Head/neck | 4(4) | 6(9) | 0.205 |
| - Trunk | 5(5) | 11(16) | **0.029** |
| - Perineum/genitals | 31(30) | 14(20) | 0.162 |
| - Upper extremity | 17(16) | 4(6) | **0.036** |
| - Lower extremity | 42(44) | 34(48) | 0.444 |
| - Multiple body areas involved | 4(4) | 2(3) | 1.000 |
| Estimated TBSA affected in percentages, median (IQR) ^c^ | 4(2-6) | 4(2-6) | 0.336 |
| CRP in mg/L, median (IQR) ^a^ | 304(161-398) | 274(134-383) | 0.601 |
| Heart rate in beats/minute, median(IQR) ^d^ | 100(89-110) | 105(90-120) | 0.077 |
| Systolic blood pressure in mmHg, mean(SD) ^c^ | 118(23) | 117(24) | 0.909 |
| Imaging, n(%) | 20(19) | 71(100) | **<0.001** |
| Non-contributing imaging, n(%) ^a^ | 17(85) | 46(65) | 0.104 |
| Imaging causing a change in treatment, n(%) | 0(0) | 22(31) | **0.001** |
| Sepsis upon admission, n(% ) ^e^ | 35(35) | 22(32) | 0.741 |
| LRINEC score, median(IQR) ^f^ | 8(5-9) | 6(4-8) | 0.103 |
| Base excess upon admission, median(IQR) ^g^ | -12(-39 - -3) | -9(-21 - -2) | 0.427 |
| Time presentation to surgery hours, median(IQR) ^h^ | 5.5(3-25) | 15(4.5-31.5) | **0.015** |
| Amputation, n(%) ^i^ | 15(15) | 7(10) | 0.488 |
| ICU admission, n(%) | 78(74) | 56(79) | 0.722 |
| Length of ICU stay in days, median(IQR) ^j^ | 5(1-12) | 4(2-9) | 0.871 |
| If survived, length of hospital stay in days, median(IQR) ^k^ | 27(17-49) | 22(12-42) | 0.203 |
| Mortality, n(%) | 23(22) | 14(20) | 0.851 |
| *ASA= American Society for Anesthesiologists, CRP = C-reactive protein, GAS= Group A Streptococcus, ICU = Intensive Care Unit, IQR= Interquartile range, LRINEC= Laboratory risk indicator for necrotizing fasciitis, NSTI= Necrotizing soft tissue infection, TBSA= Total body surface area, ^a^: 5 missings, ^b^: 8 missings,  ^c^: 9 missings, ^d^: 7 missings, ^e^: 8 missings, ^f^: 34 missings, ^g^: 80 missings, ^h^: 13 missings, ^i^: 4 missings, ^j^:23 missings, ^k^:1 missing* | | | |
